# Rime Priming Effects in Spoken Word Recognition: Are They Really Due to the Rime?

**DOI:** 10.1027/1618-3169/a000598

**Published:** 2024-01-30

**Authors:** Sophie Dufour, Jonathan Mirault, Jonathan Grainger

**Affiliations:** ^1^ CNRS, LPL, UMR 7309, Aix-Marseille Université, Aix-en-Provence, France; ^2^ Institute for Language, Communication, and the Brain, Aix-Marseille Université, Aix-en-Provence, France; ^3^ Laboratoire de Psychologie Cognitive, Aix-Marseille Université & CNRS, Aix-en-Provence, France; ^4^ Pôle pilote AMPIRIC, Institut National Supérieur du Professorat et de l'Éducation (INSPÉ), Aix-Marseille Université, Aix-en-Provence, France

**Keywords:** spoken word recognition, final overlap priming, position-independent phonemes, TISK model

## Abstract

**Abstract:** In this study, we re-examined the facilitation that occurs when auditorily presented monosyllabic primes and targets share their final phonemes, and in particular the rime (e.g., /vɔʀd/–/kɔʀd/). More specifically, we asked whether this rime facilitation effect is also observed when the two last consonants of the rime are transposed (e.g., /vɔʀd/–/kɔʀd/). In comparison to a control condition in which the primes and the targets were unrelated (e.g., /pylt/–/kɔʀd/), we found significant priming effects in both the rime (/vɔdʀ/–/kɔʀd/) and the transposed-phoneme “rime” /vɔdʀ/–/kɔʀd/ conditions. We also observed a significantly greater priming effect in the former condition than in the latter condition. We use the theoretical framework of the TISK model (Hannagan et al., 2013) to propose a novel account of final overlap phonological priming in terms of activation of both position-independent phoneme representations and bi-phone representations.


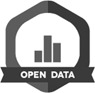


Perhaps one of the most robust effects in the literature on phonological priming during spoken word recognition is the facilitation observed on the processing of auditory target words when they are preceded by auditory primes that share their final phonemes with targets (e.g., R*AMP*–L*AMP*; Dumay et al., 2001; Monsell & Hirsh, 1998; Norris et al., 2002; Radeau, 1995; Radeau et al., 1994, 1995; Spinelli et al., 2001; Slowiaczek et al., 1987, 2000). This facilitation effect has been found to depend heavily on whether the primes and targets rhyme, and so share all phonemes from vowel to word offset. For example, in a shadowing task, Radeau (1995) manipulated the amount of final overlap between primes and targets. She observed facilitation for rime overlap (e.g., FL*AMME*–TR*AME*) in comparison to an unrelated prime condition (e.g., CLOCHE–TRAME), but no increase in the effect when the overlap included the final consonant of the onset in addition to the rime (e.g., G*RAMME*–T*RAME*). When primes and targets shared only their last consonant (e.g., FLE*MME*–TRA*ME*), no effect was found. Also, in a study using the shadowing task and controlling for the number of shared phonemes in the different priming condition, Slowiaczek et al. (2000) obtained a greater facilitation effect when primes and targets shared the rime (e.g., R*ANK*–B*ANK*) than when prime–target overlap did not include the rime (e.g., inflected words HO*NKED*–BA*NKED*). Interestingly, Slowiaczek et al. also showed that the final overlap facilitation effect was no longer observed when primes and targets shared the vowel but none of the following consonants (e.g., *RAMP–BANK*), thus suggesting that vowel overlap alone is not sufficient to obtain rime priming.

The rime priming effect has two key components that suggest that prelexical representations and not lexical representations are responsible for the effect. First, the size of rime priming effects does not vary as a function of the relative frequency of the primes and the targets. Effects of similar size were found for word targets of lower frequency than primes and for word targets of higher frequency than primes (Radeau et al., 1995). Finally, rime priming effects are not influenced by the lexical status of primes and targets. Similar effects were found with word primes and pseudoword primes (e.g., Radeau et al., 1994; Slowiaczek et al., 2000). Also, rime priming effects have been observed for word and pseudoword targets (e.g., Dumay et al., 2001). These results suggest that facilitatory rime priming effects are driven by prelexical representations that are shared across primes and targets. Activation of these shared representations during prime processing then impacts the subsequent processing of the target.

Regarding spoken word recognition models, the rime priming effect fits well with the dominant view of spoken word recognition according to which units smaller than words are extracted from the speech signal before making contact with the mental lexicon (e.g., Gaskell & Marslen-Wilson, 1997; Hannagan et al., 2013; Marslen-Wilson & Welsh, 1978; McClelland & Elman, 1986; Mehler, 1981; Norris, 1994). The rime priming effect can thus provide important evidence with respect to the nature of the prelexical representations that mediate spoken word recognition. Indeed, rime priming effects point to either subsyllabic groupings (e.g., the rime/aʀt/ in the French word “c*arte*/kaʀt/”) or simple ordered phoneme combinations such as bi-phones (e.g., the bi-phones/ka/-/aʀ/-/ʀt/ in the French word “c*arte*/kaʀt/”) that would be extracted from the speech signal and used to access lexical representations. Although units such as individual phonemes and syllables have often been proposed as intermediary units (e.g., Marslen-Wilson & Welsh, 1978; McClelland & Elman, 1986; Mehler, 1981; Norris, 1994), bi-phone representations have been envisaged only recently and are assumed in a relatively recent model of spoken word recognition, the TISK model (Time-Invariant String Kernel; Hannagan et al., 2013).

TISK is an interactive-activation model similar to the TRACE model (McClelland & Elman, 1986). Both models assume separate levels of representation for phonemes and words that are organized hierarchically, with the activation of word units determined by their degree of overlap with the activation of phoneme units. TISK shares the two key principles of the TRACE model, namely phoneme-to-word facilitation and word-to-word inhibition, but it replaces the position-dependent phoneme units in TRACE by both a set of position-independent phoneme units and a set of bi-phone units that represent ordered sequences of contiguous and noncontiguous phonemes. The position-independent phonemes and the bi-phone units postulated in TISK make it the sole model capable of accounting for a relatively new phenomenon observed in the field of spoken word recognition, the transposed-phoneme effect. This effect refers to the observation that a speech input like [kat] not only provides support for the corresponding lexical representation *cat* but also for the lexical representation that contains the same phonemes in a different order *tack* (e.g., Dufour & Grainger, 2019; Toscano et al., 2013), as well as the observation that nonwords (/ba**ks**ɛt/) created by transposing two phonemes of a real word (/ba**sk**ɛt/) are more readily confused with the base word than nonwords (/ba**pf**ɛt/) created by substituting two phonemes of the same base word (e.g., Dufour et al., 2021; Dufour & Grainger, 2022).

With respect to the aims of the present study, it is important to note that the bi-phone units postulated in TISK can also account for rime priming effects. Many studies examining rime priming effects have used rimes with a VC phonological structure (e.g., Dumay et al., 2001; Radeau, 1995; Radeau et al., 1994, 1995; Slowiaczek et al., 2000). Therefore, within the framework of TISK, it could simply be the repeated activation of the same bi-phones, composed of a vowel and a consonant, that caused the rime priming effect. Rime priming has also been reported with a more complex phonological structure and with rimes composed of a vowel and a consonantal cluster, such as in the French prime–target pair “t*arte* /taʀt/–“c*arte* /kaʀt/” (e.g., Norris et al., 2002; Slowiaczek et al., 2000). TISK can also easily account for the facilitatory priming effect observed with this type of rime. It would be due to the activation of the bi-phones /aʀ/ and /ʀt/ during the processing of the prime /taʀt/ that are then reactivated during the processing of the target /kaʀt/, thus facilitating its prelexical processing and in turn its recognition. Rimes with a VCC phonological structure are particularly relevant for the TISK model since they allow testing an important prediction of TISK that no other model of spoken word recognition makes. Due to the existence of position-independent phonemes, TISK also predicts some facilitation of processing when the target word /kaʀt/ is preceded by the word /batʀ/ that shares the same final phonemes with the target but with the two final consonants transposed. In this case, according to the TISK model, the same final position-independent phonemes /a/-/ʀ/-/t/ should be activated by both the primes and the targets, thus causing a prelexical facilitation priming effect. The present study was designed to test this important prediction.

Here, we examined whether a final overlap facilitation effect can be observed when primes and targets shared the vowel plus the final consonants in a different order, as predicted by the TISK model. To do so, CVCC monosyllabic target words were used and were preceded either by a pseudoword prime that share the final three phonemes with targets, but with the two consonants in a different order (e.g., v*odre* /vɔdʀ/–c*orde* /kɔʀd/ “rope”), or by a pseudoword “rime” prime that shared the final three phonemes in exactly the same positions as in the targets (e.g., v*orde* /vɔʀd/–c*orde* /kɔʀd/ “rope”). The magnitude of the priming effect in these two conditions was evaluated relative to a control condition where primes and targets were unrelated (e.g., pulte /pylt/–corde /kɔʀd/). The predictions were as follows. If, as postulated by the TISK model, position-independent phonemes mediate lexical processing, then we expected to find a facilitation effect when the primes and the targets share the final phonemes from the vowel but with the two final consonants transposed. We also expected to find a greater priming effect when the primes and the targets shared the final phonemes in the same positions because in this case, and within the framework of TISK, the primes and the targets have not only the final (position-independent) phonemes in common but also the final bi-phones.

## Method

### Participants

A total of 150 participants were recruited online for the experiment. All participants reported to be native speakers of French, and their reported age was between 18 and 60 years. Prior to the beginning of the experiment, participants provided informed consent and they were informed that the data would be collected anonymously. The sample size was determined on the basis of standard spoken word recognition experiments that traditionally involve between 12 and 20 participants per experimental list. Since this was an online experiment that facilitates both the recruitment of participants and running the experiment, we decided to increase the number of participants to 50 participants per experimental list. This allowed us to have a more highly powered experiment and also to ensure that enough participants remained for analysis after rejecting those with excessive error rates and/or RTs.

### Materials

Thirty-six monosyllabic target words with a CVCC syllabic structure were selected from VoCoLex, a lexical database for French (Dufour et al., 2002). For each target word, three pseudoword primes were created. The first shared the three final phonemes in the same position with the target (e.g., v*orde* /vɔʀd/–c*orde* /kɔʀd/ “rope”). The second also shared the three final phonemes but with the two final consonants transposed (e.g., v*odre* /vɔdʀ/–c*orde* /kɔʀd/). Finally, the third prime, used as control, was unrelated and shared no phonemes with the target (e.g., pulte /pylt/–corde /kɔʀd/). All the control primes had a CVCC syllabic structure. The mean frequency of the target words was 39 occurrences per million. The prime and target words are given in the Appendix. For interested readers, we also provide in the Appendix the sonority profile and the phonemic category of the last two consonants of the transposed-phoneme and rime primes.

Three experimental lists were created so that each of the 36 target words was preceded by the three types of primes (rime, transposed-phoneme, control), and participants were presented with each target word only once. For the purpose of the lexical decision task, 36 CVCC pseudowords serving as targets were created by changing either the final or the initial phoneme of words not used in the experiment (e.g., the word serpe /sɛʀp/“billhook” became /fɛʀp/). So that the pseudowords followed the same criteria as the words, 12 were paired with a pseudoword prime sharing the final phonemes in the same position (e.g., /bɛʀp/–/fɛʀp/), 12 others were paired with a pseudoword prime sharing the final phonemes but with the two final consonants transposed (e.g., /bɛpʀ/–/fɛʀp/), and the remaining 12 were paired with a pseudoword prime sharing no phonemes (e.g., /pakl/–/fɛʀp/). To achieve a low proportion of related trials (i.e., 20%), 168 unrelated prime–target pairs having no phoneme in common and serving as filler trials were added to each list. Again, for the purpose of the lexical decision task, half of the filler targets were words and the other half were pseudowords. All the filler targets were paired with a pseudoword prime. All the stimuli were recorded by a female native speaker of French, in a sound attenuated room, and digitized at a sampling rate of 44 kHz with 16-bit analog to digital recording. The mean duration of the targets words was 583 ms. The mean durations of the pseudoword primes were 580, 589, and 587, respectively, in the rime, transposed-phoneme, and control conditions.

### Procedure

The experiment was programmed using LabVanced software (Finger et al., 2017). Participants were instructed to put on their headphones and adjust the volume to a comfortable sound level. The primes and the targets were presented auditorily, and an interval (ISI) of 20 ms separated the offset of the primes and the onset of the targets. Participants were asked to make a lexical decision as quickly and accurately as possible by pressing the right arrow of their keyboard for the word response and the left arrow for the nonword response. Reaction time (RT) recording was triggered by the presentation of the target and was stopped by participants’ response. The prime–target pairs were presented randomly, and an intertrial interval of 2,000 ms elapsed between the participant’s response and the presentation of the next pair. Participants were tested on only one experimental list and began the experiment with 12 practice trials.

## Results and Discussion

Fifteen participants were excluded from the analyses. Among them, eight participants had an error rate above 40%, and the seven others had RTs greater than 1,500 ms on average. Two targets that gave rise to an error rate of more than 40% were also removed.^1^ The mean RT and percentage of correct responses to target words in each condition are presented in Table 1.

**Table 1:** Mean reaction times (in ms) and percentages of correct responses in each priming condition

	Control	Transposed	Rime
Reaction time	981	961	889
Correct responses	96	96	97

RTs on target words (available at https://osf.io/qtrp2/; Open Science Framework; Foster & Deardorff, 2017) were analyzed using linear mixed-effects models with participants and target words as crossed random factors using R software (R Development Core Team, 2016) and the *lme4* package (Baayen et al., 2008; Bates & Sarkar, 2007). The RT analysis was performed on correct responses, thus removing 181 (3.94%) data points of 4,590. RTs longer than 2,000 ms (2.74%) were considered as outliers and were excluded from the analysis. For the model to meet the assumptions of normally distributed residuals and homogeneity of variance, a log transformation was applied to the RTs (Baayen & Milin, 2010) prior to running the model. The model was run on 4,288 data points. We tested a model with the variable prime type (rime, transposed-phoneme, control) entered as fixed effect. The model failed to converge when random participant and item slopes were included (see Barr et al., 2013). Therefore, the final model only included random intercepts for participants and items.

To examine both the rime and the transposed-phoneme priming effect, we first reported the results of the model when the reference was the performance on the control primes. The model revealed a significant rime priming effect, with RTs on target words being 92 ms shorter when preceded by rime primes in comparison to control primes (β = −0.1040, *SE* = 0.0065, *t* = −15.98; *p* < .001). Crucially, the model also revealed a significant transposed-phoneme priming effect with RTs on target words being 20 ms shorter when preceded by transposed-phoneme primes in comparison to control primes (β = −0.0165, *SE* = 0.0065, *t* = −2.54; *p* = .011).

To compare the rime and the transposed-phoneme priming conditions, the model was releveled with the rime prime condition becoming the reference. The model revealed a significant difference with RTs on target words being 72 ms shorter when preceded by rime primes in comparison to transposed-phoneme primes (β = 0.0875, *SE* = 0.0065, *t* = 13.42; *p* < .001).

The percentage of correct responses was analyzed using a mixed-effects logit model (Jaeger, 2008), following the same procedure as for RTs. This analysis revealed no significant effects.

## Discussion

A large body of research on spoken word recognition using the priming paradigm has reported facilitation of processing when target words are preceded by primes that share the rime with targets (Dumay et al., 2001; Monsell & Hirsh, 1998; Norris et al., 2002; Radeau, 1995; Radeau et al., 1994, 1995; Slowiaczek et al., 2000). This facilitatory rime priming effect has been taken as evidence that units smaller than the word, and in particular the rime, play a role during spoken word recognition. Here, we reinterpret the rime priming effect within the framework of the TISK model that assumes that not only phonemes but also sequences of two phonemes (i.e., bi-phones) are extracted from the speech signal before making contact with phonological representations of words in long-term memory. Within such a framework, the rime priming effect would be due to the repeated activation of the same bi-phones during prime and target processing. Perhaps more importantly, TISK also assumes that the phonemes are coded independently of their position in the speech signal, and thus, an important prediction made by TISK is that priming effects should also occur when the phonemes that compose a rime are transposed. In accordance with this prediction, we reported a facilitation of processing when CVCC primes and targets shared their final three phonemes but with the two final consonants transposed. This transposed-rime priming effect argues in favor of the existence of position-independent phonemes. Position-independent phonemes that are activated during prime processing result in a facilitation of target processing when the targets share these position-independent phonemes.

We also observed that CVCC primes that shared the final three phonemes in the same positions as the CVCC targets, and thus the rime, generated a significantly stronger priming effect than CVCC primes that shared the three last phonemes with target words but with the two consonants in different positions. We believe that this stronger priming effect is due to the fact that when primes and targets share the “intact” rime the prime–target overlap not only involved position-independent phonemes but also the bi-phone representations as postulated in TISK. On the other hand, in the “transposed-phoneme rime” condition, the only overlap between primes and targets was at the level of position-independent phonemes. Together, our results place important constrains regarding the nature of the prelexical code that is computed during spoken word recognition and suggest that position-independent phoneme units and the combination of these phonemes (i.e., bi-phones) are extracted from the speech signal and play a key role during spoken word recognition.

At a more theoretical level, the present study provides a further demonstration in favor of a flexible coding of phoneme order in spoken word recognition. This flexibility has also been reported in the encoding of letter order in numerous studies conducted with visual words (e.g., Perea & Lupker, 2003; 2004; see Grainger, 2008, for a review) as well as in the tactile modality (Baciero et al., 2022), which like the auditory modality is inherently serial. In the modeling of word recognition, this flexibility could be achieved either by assuming uncertainty (or noise) associated with the position of phonemes/letters within words (e.g., Gomez et al., 2008) or by assuming an intermediate level of representations between the phoneme/letter and word levels composed of open bi-phones/grams (i.e., ordered sequences of contiguous and noncontiguous phonemes/letters; e.g., Grainger & van Heuven, 2004; Hannagan et al., 2013; Whitney, 2001). It should also be noted that one recent study (Harrison et al., 2020) of written language production (written and typed word production) also argues in favor of some flexibility in the representation of segment order. Hence, the flexible order-encoding of linguistic units (e.g., phonemes and letters for spoken and written word comprehension and production) would appear to be modality-independent and could thus constitute a general mechanism in language processing, which could also be applicable to a wide range of cognitive processes requiring the processing of order information (see Logan, 2021; Ordonez Magro et al., 2022).

Although the rime has often been proposed as playing a preponderant role in the final overlap priming effect, the syllable has also been proposed. For example, Dumay et al. (2001) found priming effects when primes and targets shared the complete second syllable (LU*RAGE*–TI*RAGE*) and also when they shared only the rime (LUB*AGE*–TIR*AGE*), but the facilitation was stronger in the former condition than in the latter condition. These findings have been interpreted as suggesting that both rime and syllable representations are involved in the final overlap facilitation effect. We proposed that this greater priming effect for syllable overlap than for rime overlap can be accounted for without the need to postulate syllable and rime units. Within the TISK framework, the greater priming effect seen with complete syllable overlap would be simply be due to the fact that in this condition the primes and the targets share more bi-phones than in the rime overlap condition. It remains to be explained, however, why Radeau (1995) reported no increase in priming effects when the overlap included the consonant of the onset in addition to the rime (e.g., G*RAMME*–T*RAME*), since in such a case the primes and the targets have more bi-phones in common than when the prime and the target share only the rime (e.g., FL*AMME*–TR*AME*). A possible explanation is that the final overlap facilitation effect in the onset + rime condition of Radeau (1995) was weakened by a competition process between the primes and the targets at a lexical level of processing. This reasoning is based on studies showing that the strongest competitors of a target word are those that match the target on the first phonemes (e.g., Allopenna et al., 1998; Dufour & Peereman, 2003; Slowiaczek & Hamburger, 1992) and on studies showing activation of words that mismatch on initial phonemes, at least when the mismatching phonemes have a high degree of overlap in terms of phonetic features, as in the G*RAMME*–T*RAME* pair of the study of Radeau (e.g., Allopenna et al., 1998; Connine et al., 1993; Marslen-Wilson et al., 1996).

To sum up, we reported facilitation of processing when primes and targets shared the final phonemes from the vowel to word offset, and thus, we replicated the so-called rime priming effect. Crucially, we also reported facilitation of processing when the two consonants of the “rime” are transposed. Rather than explaining the final overlap facilitation effect as reflecting the activation of syllabic representations and/or subsyllabic representations (i.e., the rime), we proposed, in accordance with the TISK model (Hannagan et al., 2013), a new account of the effect in terms of activation of both position-independent phoneme representations and bi-phone representations. More generally speaking, the results of our study provide further evidence for the role played by phoneme representations in spoken word recognition and suggest that phonemes and combinations of phonemes might suffice for accounting for various kinds of phonological priming effects.

## Footnotes

^1^
The 135 participants included in the analyses had an error rate comprised between 0% and 18%, and the 34 target words included in the analyses had an error rate comprised between 1% and 10%. The high error rate on the two target words removed from the analyses was likely due to the recordings of these stimuli that were somewhat noisy, and so not totally intelligible.

## Notes

**Open Data:** To the best of my ability and knowledge, I have provided all original materials and clear references to all other materials via a stable online repository.

The DOI pointing to the raw data underlying the findings reported in the article is as follows: https://osf.io/qtrp2/ (Dufour, 2023).

## Appendix

**Table A1:** Pseudoword primes and target words used in the experiment and sonority profile with the phonemic category of the last two consonants of the transposed-phoneme and rime primes

Control primes	Transposed-phoneme primes	Sonority profile	Rime primes	Sonority profile	Targets
Polt	rakse	P^−^ < F^−^	rasque	F^−^ > P^−^	casque
volde	fattre	P^−^ < L	farte	L > P^−^	carte
Pèfle	vattre	P^−^ < L	varte	L > P^−^	tarte
fourte	suble	P^+^ < L	sulbe	L > P^+^	bulbe
pycle	jakse	P^−^ < F^−^	jasque	F^−^ > P^−^	masque
vuste	lapre	P^−^ < L	larpe	L > P^−^	carpe
joucle	jabre	P^+^ < L	jarbe	L > P^+^	barbe
vyste	facre	P^−^ < L	farque	L > P^−^	barque
cheste	jacre	P^−^ < L	jarc	L > P^−^	parc
darme	voske	F^−^ > P^−^	vox	P^−^ < F^−^	box*
jouple	larde	L > P^+^	ladre	P^+^ < L	cadre
balque	pirgue	L > P^+^	pigre	P^+^ < L	tigre
Digle	detse	P^−^ < F^−^	deste	F^−^ > P^−^	veste
palque	bêrve	L > F^+^	bêvre	F^+^ < L	chêvre
Baste	tirffe	L > F^−^	tiffre	F^−^ < L	chiffre
Farje	vouple	P^−^ < L	voulpe	L > P^−^	poulpe
Falme	dispe	F^−^ > P^−^	dips	P^−^ < F^−^	chips*
narque	jilbe	L > P^+^	jible	P^+^ < L	cible
chalbe	lorffe	L > F^−^	loffre	F^−^ < L	coffre
Pulte	vodre	P^+^ < L	vorde	L > P^+^	corde
Saffle	sourte	L > P^−^	soutre	P^−^ < L	poutre
Farne	choulbe	L > P^+^	chouble	P^+^ < L	double
Chitre	palbe	L > P^+^	pable	P^+^ < L	fable
tourde	jalbe	L > P^+^	jable	P^+^ < L	cable
Natch	dailbe	L > P^+^	daible	P^+^ < L	faible
Barce	pilfe	L > F^−^	pifle	F^−^ < L	gifle
Jaste	voudre	P^+^ < L	vourde	L > P^+^	gourde
chuste	pavre	F^+^ < L	parve	L > F^+^	larve
Vapte	chirbe	L > P^+^	chibre	P^+^ < L	libre
berme	jitse	P^−^ < F^−^	jiste	F^−^ > P^−^	piste
chivre	vuple	P^−^ < L	vulpe	L > P^−^	pulpe
choudre	dalbe	L > P^+^	dable	P^+^ < L	sable
Parje	poulffe	L > F^−^	pouffle	F^−^ < L	souffle
farme	doulpe	L > P^−^	douple	P^−^ < L	souple
Foble	purque	L > P^−^	pucre	P^−^ < L	sucre
courde	valbe	L > P^+^	vable	P^+^ < L	table
*Note*. *target words removed from the analyses; P = plosive; F = fricative; L = liquid; ^+^ = voiced; ^−^ = unvoiced; > = superior in terms of sonority; < = inferior in terms of sonority.					
